# Modest Longitudinal Associations Between Parent-Reported Dental Fear at Age 5 and Child-Reported Dental Fear at Age 9: A FinnBrain Birth Cohort Study

**DOI:** 10.3390/dj14060344

**Published:** 2026-06-05

**Authors:** Mika Kajita, Akie Yada, Vesa Pohjola, Linnea Karlsson, Hasse Karlsson, Satu Lahti

**Affiliations:** 1 Department of Community Dentistry, Institute of Dentistry, University of Turku, 20014 Turku, Finland; vesa.pohjola@utu.fi (V.P.); satu.lahti@utu.fi (S.L.); 2 Department of Psychology and Speech-Language Pathology, University of Turku, 20014 Turku, Finland; akie.a.yada@jyu.fi; 3 The Centre of Excellence for Learning Dynamics and Intervention Research (InterLearn), University of Turku, 20014 Turku, Finland; 4 The Centre of Excellence for Learning Dynamics and Intervention Research (InterLearn), University of Jyväskylä, 40014 Jyväskylä, Finland; 5 Department of Education, University of Jyväskylä, 40014 Jyväskylä, Finland; 6 Department of Psychology, University of Jyväskylä, 40014 Jyväskylä, Finland; 7 Research Unit of Population Health, Faculty of Medicine, University of Oulu, 90570 Oulu, Finland; 8 FinnBrain Birth Cohort Study, Turku Brain and Mind Center, Department of Clinical Medicine, University of Turku, 20014 Turku, Finland; linnea.karlsson@utu.fi (L.K.); hasse.karlsson@utu.fi (H.K.); 9 Centre for Population Health Research, Turku University Hospital, University of Turku, 20520 Turku, Finland; 10 Unit of Child Psychiatry, Department of Psychiatry, Turku University Hospital, University of Turku, 20520 Turku, Finland; 11 Unit of Public Health, Department of Clinical Medicine, Turku University Hospital, University of Turku, 20520 Turku, Finland; 12 Department of Psychiatry, Turku University Hospital, University of Turku, 20520 Turku, Finland

**Keywords:** dental fear, parent report, child, longitudinal cohort, CFSS-DS-M, MDAS, feasibility

## Abstract

**Background/Objectives**: Parent report is often used to assess dental fear in early childhood, but its association with later child-reported dental fear remains unclear. This study primarily examined whether parent-reported dental fear at age 5 was associated with child-reported dental fear at age 9, and secondarily explored two options derived from the modified Children’s Fear Survey Schedule–Dental Subscale (CFSS-DS-M): a single general dental fear item and a pragmatic multi-item score. **Methods**: This secondary longitudinal observational analysis used data from the FinnBrain Birth Cohort Study. Mothers and fathers rated child dental fear at age 5 using the CFSS-DS-M. Children self-reported dental fear at age 9 using the Modified Dental Anxiety Scale (MDAS). Associations of the single item and five-item score with age-9 MDAS score were examined separately for mother- and father-reported data using Spearman correlations and adjusted linear regression models. Exploratory factor analysis examined the structure of the five-item score. **Results**: Treatment-specific CFSS-DS-M items frequently received “no experience” responses. The pragmatic five-item score showed an exploratory one-factor structure and was more feasible than the full 11-item score. Associations with age-9 dental fear were small. In adjusted analyses, both mother-reported measures were positively associated with age-9 dental fear. In father-reported data, the single item was not associated, whereas the five-item score showed a positive predictor-level association, although the adjusted model was not statistically significant. **Conclusions**: Parent-reported dental fear at age 5 provides modest information about later child-reported dental fear. In population-based cohorts in early childhood, less treatment-specific items may be useful when assessing dental fear.

## 1. Introduction

Dental fear in children is an important public health issue because it may affect later dental attendance and oral health [1,2]. A recent systematic review and meta-analysis also highlighted that dental fear and anxiety are common already in early childhood and may be related to children’s dental visit and caries experiences [3,4]. Therefore, identifying signs of dental fear early in childhood is essential. In early childhood, questionnaire-based self-report may not always be feasible because of developmental and practical constraints, and parent report is therefore often used in research and practice [5]. However, parent and child reports of child dental fear are not always concordant. Cross-sectional studies have shown only poor-to-moderate agreement between children’s self-reports and parental ratings of child dental fear [6,7]. Parents and children may also have difficulty accurately evaluating each other’s dental fear [8]. The poor agreement between parents’ and children’s ratings may partly reflect the fact that internalizing symptoms such as fear and anxiety are less directly observable to parents than overt behavioural problems [9]. Differences in parental perspectives have also been reported in other child-related domains, such as school preferences, parenting-related behaviours, and expectations for the child’s future [10,11,12]. When studying the association between a parent’s and a child’s fear, the strength of the association varied for mothers and fathers [13] and a previous FinnBrain study found that fathers and mothers did not assess child dental fear similarly [14].

However, parental ratings are sometimes necessary, as questionnaire-based self-report is generally considered more reliable from about age eight onward [15]. Although parent and child assessments are not identical, parent-reported fear in early childhood may still have value if it captures information that is associated with the child’s later self-reported dental fear.

A Finnish modified version of the Children’s Fear Survey Schedule–Dental Subscale (CFSS-DS-M) has previously been used in Finnish child and adolescent populations [16,17]. This 11-item version is a more compact, dental-treatment-focused form of the original Children’s Fear Survey Schedule and includes a single general dental fear item. Previous Finnish studies supported a two-factor structure of this modified instrument, broadly reflecting fear related to attending the dentist and fear related to treatment of dental decay [17]. However, those studies were conducted in older child samples and handled the response option “no experience of this particular matter” by recoding it as “not afraid” rather than treating it as missing. This may be problematic in young population-based cohorts, in which many children may not yet have experienced dental treatment procedures. Earlier Finnish data, for example, suggested that younger children often had very limited experience of caries treatment [18,19,20]. In the Finnish birth cohort age-5 data, our previous analysis showed that “No Experience” responses were particularly common for treatment-related items and that coding these responses as “not afraid” versus excluding them led to marked differences in mean values and interpretation, especially for invasive procedure items [14]. Thus, when the CFSS-DS-M is used in early childhood population-based research, important questions remain regarding both analysis and interpretation, such as how such data should be handled analytically, and whether early parent-reported dental fear provides useful early information about later child-reported dental fear.

Against this background, the primary aim of this study was to examine the longitudinal association between parent-reported dental fear at age 5 and child-reported dental fear at age 9. As a secondary, practice-oriented objective, we explored the feasibility of two CFSS-DS-M-derived scoring approaches in this cohort setting: a single general dental fear item and a pragmatic multi-item score from the CFSS-DS-M. The multi-item score was intended as a cohort-specific scoring approach, not as a formally validated short form of the CFSS-DS-M.

## 2. Materials and Methods

The reporting of this study was guided by the STROBE statement for observational studies (Table S1).

### 2.1. Participants

This was a secondary analysis of data from the FinnBrain Birth Cohort Study (finnbrain.fi), a multi-disciplinary cohort study on environmental and genetic factors related to child development and health [21]. Pregnant women were recruited at municipal maternity clinics in the Hospital District of Southwest Finland during 2011–2015, mainly in connection with the routine ultrasonography appointment offered at approximately gestational week 12. Mothers were also asked to invite their partners (hereafter referred to as fathers) to participate. Thus, fathers/spouses were not recruited to the same extent as mothers in the original cohort design. Fathers/spouses could be recruited if they attended the ultrasound visit or if the mother informed them about the study, but the study team had no permission to contact fathers/spouses who had not been informed or recruited. Of those informed about the study (N = 5970), 3808 mothers and 2623 fathers or other partners, expecting 3837 children, agreed to participate, and 3095 mothers and 2011 fathers returned the baseline questionnaire and entered the cohort. Participating parents provided written informed consent for their own participation and on behalf of their child. The study protocol was approved by the Ethics Committee of the Hospital District of Southwest Finland (14 June 2011; ETMK:57/180/2011 § 168).

The present study used all available data on parent-reported child dental fear at age 5, child self-reported dental fear at age 9, and the selected covariates. Because this was an available-case secondary analysis, sample sizes differed across analyses depending on variable availability and the handling of “no experience” responses in CFSS-DS-M scoring. Analytic sample sizes are therefore reported separately in the relevant tables.

### 2.2. Measures

Child dental fear at age 5 was assessed separately by mothers and fathers using a Finnish modified 11-item version of the Children’s Fear Survey Schedule–Dental Sub-scale (CFSS-DS-M) [16,17]. This more compact, dental-treatment-focused version was developed from the original CFSS-DS by excluding seven dental and medical situation items and adding items on fear of pain, suction used in the mouth, and a single general dental fear item (Item 1), in line with the single-item measure used by Milgrom et al. [22]. The CFSS-DS-M included 11 items covering general dental treatment fear, opening the mouth, the dentist, teeth cleaning, drilling, local anesthesia, sound of drilling, difficulty breathing, instruments in the mouth, suction, and pain caused by treatment. Each item was rated on a 5-point scale from 1 (not afraid) to 5 (very afraid). In the FinnBrain cohort implementation, an additional response option, “no experience of this particular matter” (coded as 6), was also available. Unlike earlier Finnish studies of the CFSS-DS-M, in which the “no experience” response was recoded as “not afraid” [17], the present study treated this category as missing in order to reflect item unavailability in this young population-based cohort.

Child dental fear at age 9 was measured using the Finnish translation of the Modified Dental Anxiety Scale (MDAS) [23,24]. The Finnish MDAS has shown validity and reliability in both adults and children aged 9–12 years [25]. The MDAS contains five items covering anticipatory and treatment-related dental fear, each rated from 1 (not anxious) to 5 (extremely anxious), yielding a total score ranging from 5 to 25. In the present study, the MDAS total score at age 9 was used as the main outcome.

In this manuscript, we use “dental fear” as an umbrella term for the construct assessed by the CFSS-DS-M and MDAS. Although dental fear and dental anxiety can be conceptually distinguished, these terms are often used together or partly interchangeably in the pediatric dental literature [26]. The term “dental fear” was used for consistency, while original instrument names, including the Modified Dental Anxiety Scale, were retained.

Child sex and parental dental fear were included as covariates based on the previous literature [26,27,28], and parental education was included as a socio-economic covariate as previous studies have suggested that family background factors may be relevant to children’s dental fear, although findings have been inconsistent [18,29,30]. Maternal education and dental fear were used in mother-reported models and paternal education and dental fear in father-reported models. Educational information was obtained from the baseline questionnaire from gestational week 14 and supplemented with age-5 data when necessary. We did not include detailed child dental experience variables, such as dental treatment, dental pain, or caries-related factors, in the main adjusted models. The aim of this study was not to establish causal mechanisms or to develop a comprehensive prediction model, but to examine the longitudinal association between age-5 parent-reported dental fear and age-9 child-reported dental fear. Therefore, the main models were kept deliberately simple and adjusted only for selected background covariates.

### 2.3. Statistical Analysis

Responses coded as “no experience” were treated as missing throughout the analyses and were not imputed. This decision was made because “no experience” was interpreted as indicating item non-applicability rather than a low level of fear or randomly missing information. In this study, the high frequency of such responses was not treated only as a missing-data problem, but as part of the practical feasibility issue under investigation. Therefore, multiple imputation was not used for CFSS-DS-M items. Alternative computed scoring approaches, such as recoding “no experience” as “not afraid”, imputing item or person means, or using peak item values, were not performed because these approaches would have mixed dental fear with treatment exposure or item applicability. In this young population-based cohort, such alternatives would have changed the construct being measured rather than provided a neutral test of the scoring decision. Instead, the availability of each item was described, and a pragmatic multi-item score was explored as a practical scoring approach using items that were less treatment-specific. As an exploratory complete-response check, we additionally examined Spearman correlations using the pragmatic five-item score calculated only from parent reports with complete data on all five selected items, and the full 11-item score calculated only from parent reports with complete data on all 11 items.

Descriptive statistics were used to summarize sample characteristics and study variables. To assess the usability of age-5 parent-reported CFSS-DS-M items, we calculated the frequency of the response option “no experience” (category 6) for each item separately for mothers and fathers. Item selection for the multi-item score was based pragmatically on the frequency distributions of these responses in both mother- and father-reported data, together with consideration of item content and the aim of retaining fewer treatment-specific items. Responses coded as “no experience” were treated as missing throughout the analyses and were not imputed, because the aim was to evaluate the practical usability of parent-reported CFSS-DS-M items in this young population-based cohort under observed response patterns.

Mother- and father-reported data were analyzed separately because a previous item-level analysis of the same FinnBrain age-5 data showed poor concordance between mothers’ and fathers’ assessments of child dental fear (Kappa range 0.072–0.258) [14]. Therefore, rather than combining parental ratings into a single averaged measure, maternal and paternal reports were modelled separately in the present study. Two age-5 parent-reported measures of child dental fear were used as alternative main predictors of the age-9 MDAS total score: a single general dental fear item and a pragmatic multi-item score. The single item was retained because general dental fear has previously been used as a separate single-question measure [31], whereas the pragmatic multi-item score was explored as a potentially more informative but still feasible indicator in this young cohort.

A pragmatic multi-item score was derived from the selected items from CFSS-DS-M based on item usability, content relevance, and the aim of retaining less treatment-specific items, and was calculated as a mean score when sufficient item-level data were available. Because these items were selected pragmatically rather than as an established short form, their structure was examined separately for mother- and father-reported CFSS-DS-M data. Exploratory factor analysis with principal axis factoring and a one-factor solution was used to assess whether they showed a one-factor structure consistent with their pragmatic summarization as a pragmatic multi-item score. Sampling adequacy and factorability were assessed using the Kaiser–Meyer–Olkin measure and Bartlett’s test of sphericity. Internal consistency of the selected items was assessed using Cronbach’s α.

Associations between age-5 CFSS-DS-M measures and age-9 child dental fear were examined using Spearman’s rank correlation coefficients because the distributions of the CFSS-DS-M measures and MDAS scores were skewed. As an exploratory complete-response check, we also examined Spearman correlations using the five-item score calculated only from parent reports with complete data on all five selected items, and the full 11-item score calculated only from parent reports with complete data on all 11 items. Multiple linear regression models were then fitted with the child’s age-9 MDAS total score as a continuous outcome to estimate adjusted associations with the age-5 parent-reported measures. The main predictors were either the age-5 single general dental fear question or the pragmatic multi-item score, analyzed separately for mother- and father-reported data. Models were adjusted for child sex, parental education, and parental MDAS total score at child aged 2. Exploratory factor analyses and regression models were based on complete cases for the variables included in each analysis. Statistical significance was set at *p* < 0.05. All analyses were conducted using IBM SPSS Statistics version 27 (IBM Corp., Armonk, NY, USA).

## 3. Results

### 3.1. Sample Characteristics

Sample characteristics are shown in Table 1. In total, 1911 children contributed data to at least one study variable used in this study. The child-reported MDAS total score at age 9, used as the main outcome, was available for 778 children. Age-5 mother-reported CFSS-DS-M items were available for 1470–1489 children, and age-5 father-reported CFSS-DS-M items were available for 706–716 children before treating “no experience” responses as missing. Thus, analytic sample sizes varied across measures because of variable availability and the handling of “no experience” responses in CFSS-DS-M scoring.

### 3.2. Availability of CFSS-DS-M Items and Feasibility of a Pragmatic Five-Item Score

The availability of CFSS-DS-M items at age 5 differed markedly across items (Table 2). Items reflecting more general dental fear had relatively few “no experience” responses, whereas several treatment-specific items showed high proportions of such responses. On this basis, items 1, 2, 3, 4, and 9 were selected for a pragmatic five-item score used in the subsequent analyses.

Exploratory factor analysis supported summarizing the selected five items as one pragmatic score in both mother- and father-reported data. In mother-reported data, the Kaiser–Meyer–Olkin measure was 0.888 and Bartlett’s test of sphericity was significant (χ^2^(10) = 3326.91, *p* < 0.001). Corresponding values for father-reported data were 0.888 and χ^2^(10) = 1326.89, *p* < 0.001. Internal consistency of the selected five-item score was high in both mother-reported (Cronbach’s α = 0.93) and father-reported data (Cronbach’s α = 0.92). Factor loadings, communalities, eigenvalues, and explained variance are shown in Table 3.

The pragmatic five-item score was also substantially more feasible than the full 11-item score. The full 11-item score was retained as a strict complete-case comparison, whereas the pragmatic five-item score was calculated pragmatically as a mean score when at least three of the five selected items were available. Among parent-specific CFSS-DS-M respondents, the full 11-item score could be calculated for only 7.6% of mother reports and 12.8% of father reports, whereas the pragmatic five-item score could be calculated for 93.6% and 88.5%, respectively. When expressed against the total analytic sample, the pragmatic five-item score was available for 72.9% of mother reports and 33.2% of father reports, compared with fewer than 6% for the full 11-item score (Table 4).

The pragmatic five-item score was calculated when at least three of five items were available; the full 11-item score required complete data on all 11 items.

### 3.3. Associations Between Age-5 Parent-Reported and Age-9 Child-Reported Dental Fear

As shown in Table 5, both the mother-reported single general dental fear item (CFSS-DS-M Item 1) and the mother-reported pragmatic five-item score were positively but weakly correlated with age-9 MDAS total score. In father-reported data, the single general dental fear item was not significantly correlated with age-9 MDAS total score, whereas the pragmatic five-item score showed a weak positive correlation.

Correlations among the age-5 maternal and paternal single-item and pragmatic five-item CFSS-DS-M measures and child MDAS total score at age 9 are shown in Table S2. The maternal and paternal measures were moderately correlated across informants, whereas the single-item and pragmatic five-item measures were strongly correlated within each informant.

The adjusted regression models showed a similar pattern (Table 6 and Table 7). In mother-reported data, both the single general dental fear item and the pragmatic five-item score were positively associated with age-9 MDAS total score after adjustment for covariates. In father-reported data, the single general dental fear item was not associated with age-9 MDAS total score. The pragmatic five-item score showed a positive association, but the overall adjusted model was not statistically significant. These father-reported regression models were based on smaller complete-case samples than the corresponding mother-reported models (N = 188–198 vs. N = 418–447).

Exploratory complete-response checks showed broadly similar weak positive correlations for the five-item score, while the full 11-item complete score was available for very few children with age-9 MDAS data (Table S3).

## 4. Discussion

In this population-based longitudinal cohort study, the primary finding was that parent-reported dental fear at age 5 showed only modest associations with child self-reported dental fear at age 9. These associations were more consistent in mother-reported than father-reported data. In mother-reported data, both the single general dental fear item and the pragmatic five-item score were associated with later MDAS total score, whereas in father-reported data only the five-item score was significantly associated with later MDAS total score, although the adjusted overall model was not statistically significant. A secondary, practice-oriented finding was that the pragmatic five-item score was substantially more feasible than the full 11-item CFSS in this early childhood cohort setting.

The modest size of the association suggests that parent report at age 5 captured only part of later child-reported dental fear. The small correlations and low R^2^ values indicate that parent-reported dental fear at age 5 had limited stand-alone predictive value for child-reported dental fear at age 9. This should not be interpreted as showing that parent reports are uninformative, but rather that they are unlikely to function as strong individual-level predictors when used alone. One possible explanation is that dental fear may change between ages 5 and 9. Previous longitudinal research has shown that parent-reported dental anxiety increased at the population level from age 5 to age 9, while also showing substantial individual-level change: many children who were anxious at age 5 were no longer anxious at age 9, whereas other children developed anxiety during follow-up [32]. During this period, children may gain new dental experiences, as caries experience appears to increase from early to middle childhood in Finnish children [18], may develop cognitively and emotionally, and may also change in terms of how they understand and express dental fear. For this reason, parent-reported dental fear at age 5 would not necessarily be expected to correspond strongly to child self-reported dental fear four years later. Rather than indicating a stable one-to-one relationship, the present findings may reflect a limited longitudinal continuity.

Another possible explanation is that parent-reported dental fear may reflect not only the child’s internal experience but also the parent’s interpretation, shaped by observable behaviour as well as the parent’s own experiences and expectations. A recent Brazilian birth-cohort study illustrates this measurement issue: dental fear in 4-year-old children was assessed by caregiver report using a single question asking whether the child was or would be afraid of going to the dentist, although most children had never visited a dentist [33]. This highlights a broader challenge in early childhood research, as caregiver-reported dental fear may partly capture anticipated dental fear in children with limited or no direct dental experience, rather than only capturing fear based on the child’s own dental experiences. Previous studies have shown poor agreement between children’s self-reports and parental proxy reports of dental fear, and parents and children may not reliably recognize each other’s dental fear [6,7,34]. More broadly, agreement between different informants on children’s internalizing symptoms is typically only modest, which may partly reflect differences in perspective and measurement across raters [9,35].

In the present study, mother-reported child dental fear showed a more consistent association with later child-reported dental fear, whereas in the father-reported data, the single general dental fear item was not associated with age-9 MDAS total score, and the father-reported pragmatic five-item score showed only a weak association at the predictor level. However, the father-reported findings should be interpreted cautiously. Although the pragmatic five-item score was significantly associated with age-9 MDAS total score at the predictor level, the overall adjusted father-reported model was not statistically significant and explained only a very small proportion of the variance. Thus, this finding should be interpreted as weak and exploratory rather than as robust evidence of a consistent father-reported association. This uncertainty may partly reflect the smaller paternal analytic sample, which was influenced by the original recruitment design because fathers/spouses were not recruited to the same extent as mothers. The smaller sample resulted in lower statistical power.

A secondary finding of this study was the marked difference in the feasibility between the full 11-item CFSS and the pragmatic five-item score. The five-item score was not intended to represent a formally validated short form of the CFSS-DS-M, but rather a cohort-specific scoring approach derived from observed item availability and item content in this early childhood population-based setting. Several CFSS items refer to specific dental procedures, such as drilling, injections, or pain during treatment. In population-based studies of young children, many children may not yet have experienced such procedures, which makes complete scoring difficult. In the present cohort, this was clearly reflected in the low feasibility of the full 11-item score. In contrast, the pragmatic five-item score could be calculated for a much larger proportion of the sample. Although the exploratory factor analysis and Cronbach’s α values supported the internal structure and consistency of the selected five items in this cohort, these findings should not be interpreted as formal validation of a short form of the CFSS-DS-M. Rather, they suggest that the pragmatic five-item score may be a useful practical scoring approach in early childhood population-based research.

The single general dental fear item is also worth noting. It had the highest practical availability and, in mother-reported data, showed an association with later child-reported dental fear that was similar in magnitude to that of the five-item score. A separate single-question measure of general dental fear has also been used in earlier Finnish studies, suggesting there is some precedent for considering such a pragmatic indicator alongside multi-item summaries [31,34]. However, the evidence for its use as a stand-alone indicator remained limited, as this association was not observed in father-reported data and a single item cannot provide information on internal consistency or factor structure. In addition, the five-item score may have captured variation in early dental fear more effectively, because averaging across five items provides a more continuous measure, whereas the single general dental fear item was based on a single five-point response. Thus, the item may be useful as a very pragmatic indicator, but the five-item score appears to provide stronger support as a short, parent-reported approach in early childhood population-based research.

The present findings also have implications for future research. First, to better understand whether the modest longitudinal associations observed here reflect a true developmental change in dental fear, future studies should include repeated assessments across childhood. Second, studies would benefit from using developmentally appropriate self-report measures in early childhood, such as face-based scales, so that the child’s own experience can be captured earlier and not only through parent report [5]. Third, more advanced analytic approaches may help separate child fear from parent-related reporting tendencies. For example, structural equation models including parental method factors may clarify the extent to which parent ratings reflect the child’s fear versus parent-specific bias.

This study has several strengths, including its population-based longitudinal design, the separate examination of mother- and father-reported dental fear, and the use of child self-reported dental fear at age 9 as the outcome. At the same time, some limitations should be noted. The observed associations were small, and the pragmatic five-item score was explored as a practical approach rather than established as a formally validated short form. In addition, the “no experience” option reflected a cohort-specific practical adaptation rather than a standard feature of the original CFSS-DS. Missing data were a major limitation. Of the 1911 children who contributed data to at least one study variable, only 778 had an age-9 MDAS total score, and the complete-case regression samples were smaller, particularly in the father-reported models. For father-reported data, the smaller sample also reflected the original recruitment design, as fathers/spouses were not recruited to the same extent as mothers. Because no information was available on fathers/spouses who were not recruited, we could not examine how they differed from recruited or responding fathers/spouses. Therefore, selection bias due to missing data and differential data availability cannot be excluded. Families and fathers with complete data or recruited/responding fathers may have differed from those without complete data or father-reported data in parental involvement, socio-economic background, child dental care experiences, or dental fear. This selection could have attenuated, inflated, or otherwise altered the observed associations. In addition, the smaller father-reported analytic samples reduced statistical power and increased the uncertainty of the paternal estimates. Therefore, the father-reported findings should be interpreted cautiously and should not be taken as strong evidence for either the presence or absence of paternal associations. Finally, the findings are based on a Finnish population-based birth cohort, and their generalizability to other cultural, healthcare, or clinical settings remains to be examined. Future studies designed to examine developmental mechanisms of dental fear more directly should incorporate detailed child dental experience variables and analytic approaches that account for parent-related reporting factors.

## 5. Conclusions

In this population-based cohort study, parent-reported dental fear at age 5 was associated with child self-reported dental fear at age 9, although the association was small. The findings suggest that parent-reported dental fear in early childhood provided only limited information about later child-reported dental fear and had limited stand-alone predictive value. Findings from father-reported models should be interpreted particularly cautiously, as the overall adjusted paternal model was not statistically significant, and the paternal analytic sample was limited. In this population-based sample of 5-year-old children, treatment-specific CFSS items were practically difficult to use, whereas a pragmatic five-item score based on fewer treatment-specific items was more feasible in this cohort setting. These findings support the practical value of parent report in early childhood population-based research, while also highlighting the need for future studies using early child self-report measures and analytic approaches that better account for parent-related reporting bias.

## Figures and Tables

**Table 1:** Sample characteristics and descriptive statistics of key study variables.

Total Sample (N = 1911)			
Variable	N (%)	Mean (SD)	Range
Child sex, n (%)			
Boy	1002 (52.4)		
Girl	909 (47.6)		
Maternal education, n (%)			
Mid/low (1–5)	504 (26.4)		
High/vocational (6)	566 (29.6)		
High (7–9)	795 (41.6)		
Missing	46 (2.4)		
Paternal education, n (%)			
Mid/low (1–5)	499 (26.1)		
High/vocational (6)	375 (19.6)		
High (7–9)	383 (20.0)		
Missing	654 (34.2)		
Mother-reported MDAS total at child’s age 2	1210	10.48 (4.53)	5–25
Father-reported MDAS total at child’s age 2	611	8.88 (3.74)	5–25
**Child dental fear**			
MDAS total at age 9	778	12.49 (4.46)	5–25
MDAS item 1 at age 9	779	1.78 (0.96)	1–5
MDAS item 2 at age 9	775	1.84 (0.95)	1–5
MDAS item 3 at age 9	776	3.36 (1.38)	1–5
MDAS item 4 at age 9	778	2.79 (1.35)	1–5
MDAS item 5 at age 9	777	2.71 (1.25)	1–5

SD = standard deviation. Ns vary across variables because of missing data.

**Table 2:** Availability of CFSS-DS-M items and frequency of “no experience” responses at age 5.

CFSS-DS-M Item	Mother Valid n	Mother “No Experience” n (%)	Father Valid n	Father “No Experience” n (%)	Included in Pragmatic Five-Item Score
Item 1. General dental fear	1488	31 (2.1)	716	38 (5.3)	Yes
Item 2. Opening mouth	1488	37 (2.5)	714	57 (8.0)	Yes
Item 3. Dentist	1489	143 (9.6)	716	85 (11.9)	Yes
Item 4. Teeth cleaning	1483	501 (33.8)	714	232 (32.5)	Yes
Item 5. Drilling	1473	1327 (90.1)	711	600 (84.4)	No
Item 6. Injection	1474	1287 (87.3)	707	567 (80.2)	No
Item 7. Sound of drill	1472	1306 (88.7)	710	597 (84.1)	No
Item 8. Difficulty breathing	1470	1195 (81.3)	706	518 (73.4)	No
Item 9. Instruments in mouth	1474	395 (26.8)	707	230 (32.5)	Yes
Item 10. Suction	1477	825 (55.9)	709	391 (55.1)	No
Item 11. Pain caused by treatment	1475	1260 (85.4)	709	550 (77.6)	No

Note. “Valid n” indicates the number of non-missing responses for the original item. “No experience” indicates the number and percentage of responses in category 6, calculated among valid responses.

**Table 3:** Exploratory one-factor structure of the pragmatic five-item score in mother- and father-reported CFSS-DS-M.

Item	Mother Loading	Mother Communality	Father Loading	Father Communality
Item 1. General dental fear	0.851	0.725	0.829	0.687
Item 2. Opening mouth	0.808	0.653	0.792	0.627
Item 3. Dentist	0.860	0.740	0.853	0.728
Item 4. Teeth cleaning	0.914	0.836	0.888	0.788
Item 9. Instruments in mouth	0.853	0.728	0.788	0.620

Mother-reported model: N = 807; Eigenvalue of factor 1 = 3.943; variance explained = 73.7%. Father-reported model: N = 386; Eigenvalue of factor 1 = 3.756; variance explained = 69.0%. Extraction method: principal axis factoring. One factor was extracted in both mother- and father-reported models.

**Table 4:** Availability and feasibility of full 11-item and pragmatic five-item CFSS-DS-M scoring approaches.

Parent Report	CFSS-DS-M Respondents n/Total Sample	Scoring Approach	Scorable n/CFSS Respondents	Scorable n/Total Sample
Mother	1489/1911 (77.9%)	Full 11-item complete score	113/1489 (7.6%)	113/1911 (5.9%)
Mother	1489/1911 (77.9%)	Pragmatic five-item score	1393/1489 (93.6%)	1393/1911 (72.9%)
Father	716/1911 (37.5%)	Full 11-item complete score	92/716 (12.8%)	92/1911 (4.8%)
Father	716/1911 (37.5%)	Pragmatic five-item score	634/716 (88.5%)	634/1911 (33.2%)

**Table 5:** Spearman correlations of age-5 CFSS-DS-M measures with age-9 MDAS total scores.

Age-5 CFSS-DS-M Measure	MDAS Total at Age 9
Mother-reported single general dental fear item	0.122 ** (n = 584)
Mother-reported pragmatic five-item score	0.108 * (n = 552)
Father-reported single general dental fear item	0.067 (n = 291)
Father-reported pragmatic five-item score	0.137 * (n = 278)

Note. Values are Spearman’s rho. * *p* < 0.05, ** *p* < 0.01.

**Table 6:** Multiple regression models predicting age-9 MDAS total score using CFSS item 1.

Predictor	Mother Model			Father Model		
	B (SE)	β	*p*	B (SE)	β	*p*
Single general dental fear item at age 5	0.698 (0.265)	0.125	0.009	0.451 (0.416)	0.080	0.279
Child sex (1 = boy; 2 = girl)	1.292 (0.423)	0.144	0.002	0.516 (0.660)	0.057	0.435
Parental MDAS total at age 2	−0.003 (0.050)	−0.003	0.952	−0.064 (0.091)	−0.052	0.481
Parental education	0.075 (0.267)	0.013	0.778	−0.245 (0.401)	−0.044	0.543
Constant	9.295 (1.044)	—	<0.001	11.696 (1.665)	—	<0.001

Mother model: N = 447; R^2^ = 0.040; adjusted R^2^ = 0.031; F = 4.583; *p* = 0.001. Father model: N = 198; R^2^ = 0.014; adjusted R^2^ = −0.006; F = 0.689; *p* = 0.600.

**Table 7:** Multiple regression models predicting age-9 MDAS total score using the pragmatic five-item score.

Predictor	Mother Model			Father Model		
	B (SE)	β	*p*	B (SE)	β	*p*
Pragmatic five-item score at age 5	0.796 (0.299)	0.132	0.008	1.073 (0.506)	0.159	0.035
Child sex (1 = boy; 2 = girl)	1.246 (0.440)	0.138	0.005	0.453 (0.675)	0.050	0.503
Parental MDAS total at age 2	−0.002 (0.052)	−0.002	0.969	−0.064 (0.094)	−0.050	0.495
Parental education	0.049 (0.280)	0.008	0.862	−0.143 (0.408)	−0.026	0.726
Constant	9.346 (1.098)	—	<0.001	10.473 (1.791)	—	<0.001

Mother model: N = 418; R^2^ = 0.039; adjusted R^2^ = 0.030; F = 4.177; *p* = 0.003. Father model: N = 188; R^2^ = 0.032; adjusted R^2^ = 0.011; F = 1.521; *p* = 0.198.

## Data Availability

The datasets presented in this article are not readily available due to restrictions imposed by the EU General Data Protection Regulation (GDPR) and local legislation. Requests to access the datasets should be directed to L.K.

## Supplementary Materials

The following supporting information can be downloaded at: https://www.mdpi.com/article/10.3390/dj14060344/s1, Table S1: STROBE checklist; Table S2: Spearman correlations among age-5 parent-reported CFSS-DS-M measures and child MDAS total score at age 9; Table S3: Exploratory complete-response Spearman correlations between age-5 parent-reported CFSS-DS-M scores and age-9 child-reported MDAS total score.

## Abbreviations

The following abbreviations are used in this manuscript:

CFSS-DS-M	modified Children’s Fear Survey Schedule–Dental Subscale
MDAS	Modified Dental Anxiety Scale
