# Supplementary material for: Modest Longitudinal Associations Between Parent-Reported Dental Fear at Age 5 and Child-Reported Dental Fear at Age 9: A FinnBrain Birth Cohort Study
Source: Dent J (Basel). 2026 Jun 5;14(6):344. doi: 10.3390/dj14060344 (PMC13298212; doi:10.3390/dj14060344)
Supplement: Supplementary file 1 [file dentistry-14-00344-s001.zip › Table S1. STROBE_checklist.pdf]

STROBE Statement—checklist of items that should be included in reports of observational studies

	Item No.	Recommendation	Page No.	Relevant text from manuscript
**Title and abstract**	1	(*a*) Indicate the study's design with a commonly used term in the title or the abstract	1	Abstract: "This secondary longitudinal observational analysis used data from the FinnBrain Birth Cohort Study." Title also identifies the study as "A FinnBrain Birth Cohort Study."
		(*b*) Provide in the abstract an informative and balanced summary of what was done and what was found	1	Abstract summarizes the background/objectives, methods, results, and conclusions, including the age-5 parent-reported CFSS-DS-M measures, age-9 child-reported MDAS outcome, Spearman correlations, adjusted regression models, EFA, and the finding that associations were small.
**Introduction**				
Background/rationale	2	Explain the scientific background and rationale for the investigation being reported	2, 3	Introduction: Dental fear in children is an important public health issue because it may affect later dental attendance and oral health. Parent-report is often used in early childhood, but parent- and child-reports are not always concordant. The CFSS-DS-M includes a "no experience" option, which may be problematic in young population-based cohorts.
Objectives	3	State specific objectives, including any prespecified hypotheses	2, 3	Introduction: "Against this background, the primary aim of this study was to examine the longitudinal association between parent-reported dental fear at age 5 and child-reported dental fear at age 9. As a secondary, practice-oriented objective, we explored the feasibility of two CFSS-DS-M-derived scoring approaches in this

				cohort setting: a single general dental fear item and a pragmatic multi-item score from the CFSS-DS-M. The multi-item score was intended as a cohort-specific scoring approach, not as a formally validated short form of the CFSS-DS-M.”
**Methods**				
Study design	4	Present key elements of study design early in the paper	4, 1	Participants: "This was a secondary analysis of data from the FinnBrain Birth Cohort Study." Abstract: "This secondary longitudinal observational analysis used data from the FinnBrain Birth Cohort Study."
Setting	5	Describe the setting, locations, and relevant dates, including periods of recruitment, exposure, follow-up, and data collection	4	Participants: Pregnant women were recruited at municipal maternity clinics in the Hospital District of Southwest Finland during 2011-2015, mainly at the routine ultrasound appointment at approximately gestational week 12. The present study used parent-reported child dental fear at age 5 and child self-reported dental fear at age 9.
Participants	6	(*a*) *Cohort study*—Give the eligibility criteria, and the sources and methods of selection of participants. Describe methods of follow-up<br>*Case-control study*—Give the eligibility criteria, and the sources and methods of case ascertainment and control selection. Give the rationale for the choice of cases and controls<br>*Cross-sectional study*—Give the eligibility criteria, and the sources and methods of selection of participants	4	Participants: Pregnant women were recruited at municipal maternity clinics in the Hospital District of Southwest Finland during 2011–2015. Mothers were asked to invite their partners to participate. Fathers/spouses were not recruited to the same extent as mothers; they could be recruited if they attended the ultrasound visit or if the mother informed them about the study. Of those informed about the study, 3808 mothers and 2623 fathers or other partners agreed to participate, and 3095 mothers and 2011 fathers

				returned the baseline questionnaire and entered the cohort. The present study used all available data on age-5 parent-reported dental fear, age-9 child-reported dental fear, and selected covariates.
		(*b*) *Cohort study*—For matched studies, give matching criteria and number of exposed and unexposed<br>*Case-control study*—For matched studies, give matching criteria and the number of controls per case	–	Not applicable. This was not a matched cohort study and no exposed/unexposed matching was used.
Variables	7	Clearly define all outcomes, exposures, predictors, potential confounders, and effect modifiers. Give diagnostic criteria, if applicable	5, 6	Measures/Statistical analysis: The outcome was age-9 child MDAS total score. The main predictors were the age-5 parent-reported single general dental fear item and the pragmatic multi-item/5-item score, analysed separately for mother- and father-reported data. Covariates were child sex, parental education, and parental MDAS total score at child age 2.
Data sources/ measurement	8*	For each variable of interest, give sources of data and details of methods of assessment (measurement). Describe comparability of assessment methods if there is more than one group	*4, 5*	Measures: Age-5 child dental fear was assessed separately by mothers and fathers using the Finnish modified 11-item CFSS-DS-M. Age-9 child dental fear was measured using the Finnish MDAS. Education was obtained from the baseline questionnaire at gestational week 14 and supplemented with age-5 data when necessary.
Bias	9	Describe any efforts to address potential sources of bias	5	Methods/Discussion: Potential sources of bias were addressed by treating “no experience” responses as missing rather than as low fear, by analysing mother- and father-reported data separately because of previously reported poor concordance, and by adjusting regression

				models for child sex, parental education, and parental MDAS at child age 2. The manuscript also discusses potential selection bias due to missing data and differential father-reported data availability, noting that fathers/spouses were not recruited to the same extent as mothers.
Study size	10	Explain how the study size was arrived at	4, 6	Participants: "The present study used all available data on parent-reported child dental fear at age 5, child self-reported dental fear at age 9, and the selected covariates." In total, 1911 children contributed data to at least one study variable; analytic sample sizes differed by analysis and are reported in the relevant tables.

Continued on next page

Quantitative variables	11	Explain how quantitative variables were handled in the analyses. If applicable, describe which groupings were chosen and why	4, 5	Methods: CFSS-DS-M and MDAS items were rated on 5-point scales. The pragmatic 5-item score was calculated as a mean score when at least 3 of the 5 selected items were available. Age-9 MDAS total score was analysed as a continuous outcome. Education was grouped as mid/low, high/vocational, and high.
Statistical methods	12	(*a*) Describe all statistical methods, including those used to control for confounding	5, 6	Statistical analysis: Descriptive statistics summarized sample characteristics and study variables. “No experience” frequencies were calculated for each CFSS-DS-M item. EFA with principal axis factoring and a one-factor solution examined the pragmatic 5-item score. Spearman correlations were used because distributions were skewed. Multiple linear regression estimated adjusted associations. Models adjusted for child sex, parental education, and parental MDAS total score at child age 2. As an exploratory complete-response check, Spearman correlations were also examined using the 5-item score calculated only from complete 5-item responses and the full 11-item score calculated only from complete 11-item responses. Analyses used IBM SPSS Statistics version 27.
		(*b*) Describe any methods used to examine subgroups and interactions	–	Not applicable. No subgroup or interaction analyses were performed.

			Mother- and father-reported data were analyzed separately as prespecified parallel reporter-specific analyses, not as subgroup interaction tests.
	(*c*) Explain how missing data were addressed	5, 6	Statistical analysis: Responses coded as “no experience” were treated as missing throughout the analyses and were not imputed, because they were interpreted as item non-applicability rather than low fear or randomly missing information. Multiple imputation was not used because the high frequency of such responses was treated as part of the feasibility issue under investigation. Alternative scoring approaches, such as recoding “no experience” as “not afraid” or imputing item/person means, were not performed. EFA and regression models were based on complete cases for the variables included in each analysis.
	(*d*) *Cohort study*—If applicable, explain how loss to follow-up was addressed<br>*Case-control study*—If applicable, explain how matching of cases and controls was addressed<br>*Cross-sectional study*—If applicable, describe analytical methods taking account of sampling strategy	4	Cohort study: Loss to follow-up was addressed descriptively through reporting data availability and analysis-specific sample sizes. Participants: the age-9 MDAS total score was available for 778 children; age-5 mother-reported CFSS-DS-M items for 1470-1489 children; age-5 father-reported items for 706-716 children before treating "no experience" as missing.
	(*e*) Describe any sensitivity analyses	–	As an exploratory complete-response

				check, Spearman correlations were examined using the pragmatic 5-item score calculated only from parent reports with complete data on all 5 selected items, and the full 11-item score calculated only from parent reports with complete data on all 11 items. These results are reported in Table S3.
**Results**				
Participants	13*	(a) Report numbers of individuals at each stage of study—eg numbers potentially eligible, examined for eligibility, confirmed eligible, included in the study, completing follow-up, and analysed	4, 6, 9	Participants/Results: Of N = 5970 informed, 3808 mothers and 2623 fathers/partners agreed to participate; 3095 mothers and 2011 fathers entered the cohort. The total study sample included 1911 children, although analytic sample size varied across measures because of missing data. Regression models reported analysis-specific N values in Tables 6 and 7.
		(b) Give reasons for non-participation at each stage	6	Participants/Results: Reasons for non-participation at each stage were not reported in detail. Data availability varied across variables, and missingness is described in the Participants section and Table 1.
		(c) Consider use of a flow diagram	–	No flow diagram was included. Participant numbers and analysis-specific sample sizes are described in the text and tables.
Descriptive data	14*	(a) Give characteristics of study participants (eg demographic, clinical, social) and information on exposures and potential confounders	6	Results/Table 1: Sample characteristics are shown for the total sample (N =

				1911), including child sex, maternal and paternal education, parental MDAS total scores at child age 2, and child MDAS item and total scores at age 9.
		(b) Indicate number of participants with missing data for each variable of interest	6	Results/Table 1: Missing data are shown for education and Ns are provided for parental MDAS and child MDAS variables. Participants section reports data availability for child sex, age-9 MDAS total score, and age-5 mother- and father-reported CFSS-DS-M items.
		(c) *Cohort study*—Summarise follow-up time (eg, average and total amount)		Cohort study: The relevant follow-up interval was from parent-reported dental fear at child age 5 to child self-reported dental fear at age 9, i.e., approximately four years.
Outcome data	15*	*Cohort study*—Report numbers of outcome events or summary measures over time	*6*	Outcome data: The main outcome was MDAS total score at age 9. Table 1 reports MDAS total at age 9: N = 778, mean = 12.49, SD = 4.46, range = 5-25, and MDAS item-level summary measures.
		Case-control study—Report numbers in each exposure category, or summary measures of exposure	–	Not applicable. This was not a case-control study.
		Cross-sectional study—Report numbers of outcome events or summary measures	–	Not applicable. This was not a cross-sectional study.
Main results	16	(*a*) Give unadjusted estimates and, if applicable, confounder-adjusted estimates and their precision (eg, 95% confidence interval). Make clear which confounders were adjusted for and why they were included	9, 10	Main results: Table 5 reports unadjusted Spearman correlations. Tables 6 and 7 report adjusted regression estimates with B, SE, standardized beta, and p values. Covariates were child sex,

			parental MDAS total at child age 2, and parental education.
	(*b*) Report category boundaries when continuous variables were categorized	6, 10	Results/Table 1 and regression tables: Child sex was coded 1 = boy and 2 = girl. Education was grouped as mid/low (1-5), high/vocational (6), and high (7-9). No other continuous variables were categorized.
	(*c*) If relevant, consider translating estimates of relative risk into absolute risk for a meaningful time period	–	Not applicable. Relative risks were not estimated; the outcome was continuous MDAS total score and associations were reported using correlations and linear regression coefficients.

Continued on next page

Other analyses	17	Report other analyses done—eg analyses of subgroups and interactions, and sensitivity analyses	8	Other analyses: EFA supported a one-factor structure for the pragmatic 5-item score in both mother- and father-reported data; Table 3 reports factor loadings, communalities, eigenvalues, and explained variance. Table 4 reports feasibility of full 11-item and pragmatic 5-item scoring approaches. Table S2 reports correlations among age-5 maternal and paternal measures and age-9 child MDAS total score. Table S3 reports exploratory complete-response Spearman correlations for the pragmatic 5-item score and the full 11-item score.
**Discussion**				
Key results	18	Summarise key results with reference to study objectives	10, 11	Discussion: The primary finding was that parent-reported dental fear at age 5 showed only modest associations with child self-reported dental fear at age 9. These associations were more consistent in mother-reported than father-reported data. In father-reported data, only the pragmatic 5-item score was significantly associated at the predictor level, although the adjusted overall model was not statistically significant. A secondary, practice-oriented finding was that the pragmatic 5-item score was substantially more feasible than the full 11-item CFSS in this early childhood cohort setting.

Limitations	19	Discuss limitations of the study, taking into account sources of potential bias or imprecision. Discuss both direction and magnitude of any potential bias	12	Limitations: The observed associations were small, and the pragmatic 5-item score was explored as a practical approach rather than established as a formally validated short form. The “no experience” option was cohort-specific. Missing data were a major limitation, particularly because only 778 of 1911 children had age-9 MDAS total scores and complete-case regression samples were smaller, especially in father-reported models. For father-reported data, the smaller sample also reflected the original recruitment design, as fathers/spouses were not recruited to the same extent as mothers. Selection bias due to missing data and differential data availability cannot be excluded, and such selection could have attenuated, inflated, or otherwise altered the observed associations. Generalizability beyond the Finnish birth cohort remains to be examined.
Interpretation	20	Give a cautious overall interpretation of results considering objectives, limitations, multiplicity of analyses, results from similar studies, and other relevant evidence	12–13	Discussion/Conclusions: The findings are interpreted cautiously as limited longitudinal continuity rather than a stable one-to-one relationship. Parent report may provide some information about later child-reported dental fear, but it does not fully capture the child’s later subjective experience and has limited stand-alone predictive value. Father-

				reported findings are interpreted particularly cautiously because the overall adjusted paternal model was not statistically significant and the paternal analytic sample was limited. The pragmatic 5-item score may be useful as a practical scoring approach, but not as a formally validated short form.
Generalisability	21	Discuss the generalisability (external validity) of the study results	13	Limitations: "The findings are based on a Finnish population-based birth cohort, and their generalizability to other cultural, healthcare, or clinical settings remains to be examined."
**Other information**				
Funding	22	Give the source of funding and the role of the funders for the present study and, if applicable, for the original study on which the present article is based	13	Funding: This research was funded by the Research Council of Finland, Jane and Aatos Erkko Foundation, the Academy of Finland Centre of Excellence Programme/InterLearn CoE, and the SYS-LIFE postdoctoral programme co-funded by the European Union's Horizon Europe Framework Programme under Marie Skłodowska-Curie grant agreement no. 101126611 and the University of Turku.

*Give information separately for cases and controls in case-control studies and, if applicable, for exposed and unexposed groups in cohort and cross-sectional studies.

Note: An Explanation and Elaboration article discusses each checklist item and gives methodological background and published examples of transparent reporting. The STROBE checklist is best used in conjunction with this article (freely available on the Web sites of PLoS Medicine at http://www.plosmedicine.org/, Annals of Internal Medicine at http://www.annals.org/, and Epidemiology at http://www.epidem.com/). Information on the STROBE Initiative is available at www.strobe-statement.org.
