# Supplementary material for: Modest Longitudinal Associations Between Parent-Reported Dental Fear at Age 5 and Child-Reported Dental Fear at Age 9: A FinnBrain Birth Cohort Study
Source: Dent J (Basel). 2026 Jun 5;14(6):344. doi: 10.3390/dj14060344 (PMC13298212; doi:10.3390/dj14060344)
Supplement: Supplementary file 1 [file dentistry-14-00344-s001.zip › Table S2.pdf]

Table S1. Spearman correlations among age-5 parent-reported CFSS-DS-M measures and child MDAS total score at age 9

Variable	1	2	3	4	5
1. Mother CFSS item 1 at age 5	1				
2. Mother 5-item general fear score at age 5	.862**	1			
3. Father CFSS item 1 at age 5	.424**	.434**	1		
4. Father 5-item general fear score at age 5	.472**	.482**	.872**	1	
5. Child MDAS total at age 9	.122**	.108*	0.067	.137*	1

Note. Values are Spearman's rho. Pairwise sample sizes varied because of missing data. * p < .05, ** p < .01.
