# Supplementary material for: Modest Longitudinal Associations Between Parent-Reported Dental Fear at Age 5 and Child-Reported Dental Fear at Age 9: A FinnBrain Birth Cohort Study
Source: Dent J (Basel). 2026 Jun 5;14(6):344. doi: 10.3390/dj14060344 (PMC13298212; doi:10.3390/dj14060344)
Supplement: Supplementary file 1 [file dentistry-14-00344-s001.zip › Table S3.pdf]

Table S3. Exploratory complete-response Spearman correlations between age-5 parent-reported CFSS-DS-M scores and age-9 child-reported MDAS total score

Parent report	Age-5 CFSS-DS-M measure	Scoring rule	Scorable N in total sample	N with age-9 MDAS	Spearman's rho	p
Mother	Pragmatic 5-item score	Mean score, ≥3/5 valid items	1393	552	.108	.011
Mother	Complete 5-item score	Mean score, 5/5 valid items	807	318	.114	.043
Mother	Full 11-item score	Mean score, 11/11 valid items	113	46	.093	.540
Father	Pragmatic 5-item score	Mean score, ≥3/5 valid items	634	278	.137	.023
Father	Complete 5-item score	Mean score, 5/5 valid items	386	174	.106	.163
Father	Full 11-item score	Mean score, 11/11 valid items	92	48	.084	.571

Note. Values are Spearman's rho. "No experience" responses were treated as missing. Correlation Ns differ from scorable Ns because age-9 MDAS data were also required.
